# The Implication of Digital Organisational Culture on Firm Performance

**DOI:** 10.3389/fpsyg.2022.840699

**Published:** 2022-05-12

**Authors:** Mahir Pradana, Anita Silvianita, Syarifuddin Syarifuddin, Renaldi Renaldi

**Affiliations:** ^1^Department of Business Administration, Telkom University, Bandung, Indonesia; ^2^Department of Management, Andi Djemma University of Palopo, Palopo, Indonesia

**Keywords:** organisational culture, human resource management, business administration, business digitalisation, human capital

## Abstract

Digital technologies have become a major factor for innovation in the business environment. Organisations have taken advantage of digitised data and information to increase performance. However, there is still little research focusing on the effect of digitalisation on organisational culture, which in the end will affect performance. We develop this research by exploring a proposed model involving digital organisational culture with the final goal to enhance organisational performance. The research involved 263 managers of state-owned companies in Indonesia. We analysed the theoretical model by using structural equation modelling and processed the data using the SmartPLS version 3 software. We conclude that digital organisational culture can become an essential factor in improving digital strategy and performance. However, business digitalisation does not really affect digital organisational values.

## Introduction

A thorough study has been carried out by academics related to business digitalisation in improving business performance, regardless of which object is taken as evidence, such as multinational companies (Son et al., 2020). Research conducted by these experts yielded various findings, where the same object obtains different results. Some research results state that business digitalisation influences overall performance (Pradana et al., 2020). However, there is still some research which states that business digitalisation has no direct effect on performance, also that flexible strategy is needed to win the competition, and to pay attention to the external environment (Kanwal et al., 2019).

Differences in results on performance orientation from some scholars result in many gaps which proves that the concept of business digitalisation deserves to be measured and continuously investigated (Fakhri et al., 2020). Views related to business digitalisation are much juxtaposed with the accuracy of the unit of analysis; given that view, it is more appropriate to conduct it on corporates as objects with clear insight and competence (Kartawinata et al., 2021). Employees with an excellent educational background might yield a high number of success because competitive conditions are tight, especially when equipped with tenacity in running the business and using digital technology to develop (Pradana et al., 2020). The decisions that were taken have already taken into account the impact of risk to the company and anticipatory steps to take when such inevitable loss happens. Employees must always be proactive in seeking opportunities, information, and in taking advantage of the opportunity because the advantages can only be experienced by those who act first (Birasnav et al., 2019).

Academics have begun to analyse the facts that are happening in today's world of business. Innovation is widely acknowledged as the key to a company's success in improving its business performance (Pradana et al., 2020). Good innovation can be created by giving broad autonomy to entities within the company (Abbasi et al., 2021). By granting autonomy to each individual in the company, they will be more creative in their innovations (Blouch et al., 2021). In previous studies, business digitalisation does not really affect performance (Fakhri et al., 2020). However, what makes it unique is that the treatment in improving the required performance includes several variables which are relevant to the appropriate digitalisation of the business (Abbasi et al., 2021; Devi et al., 2021).

With technology being a crucial element of business activities in the digital era, it has created an entity that can organise and design supply chain patterns along with value creation in various jurisdictions but manage to stay integrated (Hasbi et al., 2020). It is also what makes geographical boundaries unclear, unblocked, and makes the distance between areas of the economic activity in a group of business entities become increasingly meaningless (Blouch et al., 2021). The involved activities are integration of vertical relationships in the production process (vertical networking of an intelligent production system), the integration of horizontal relationships characterised by the structure of supply chain patterns (horizontal integration via a new generation of global value chain networks), the existence of mechanisation processes in the overall supply chain (through engineering across the entire value chain), and accelerated economic activity driven by technological developments (acceleration through exponential technology) (Kang and Kim, 2017).

Therefore, we are certain that we can provide a significant contribution by conducting this research. The study is structured as follows: introduction, conceptual framework and the hypotheses of the study, followed by the explanation of the methods, the results, and the conclusion and future research directions will be presented last.

## Literature Review

Nowadays, it has been widely accepted that digital technology (which is considered as a combination of information technology, connectivity, communication, computing, etc.) can be utilised in business to achieve the essence of sustainable competitive advantage for the continuity of competition in several divisions. According to Mansoor and Wijaksana (2021), digitalisation of business is a method in which companies can explore and adopt new digital technologies. Zaman et al. (2022) stated that digital formats provide convenience in various activities as it offers flexibility, reliability, and lower costs. Therefore, over the last decade, in order to improve the interconnection between products, processes, and services, many business infrastructures have been transformed into a digital format (Devi et al., 2021). Digital technology in companies of various industries and sectors can fundamentally change business processes, company capabilities, products and services, and even relationships between companies into a wider business network (Willayat et al., 2022).

Several research has discussed the digitalisation process from the operational aspects. However, organisations can facilitate the digitalisation process in different ways. The history of technological advancements in business is filled with examples of companies focusing on technology without investing in organisational capabilities that ensure their impact. In implementing technology, many companies failed because the organisation cannot change their employees' mindset and processes or efforts to build a culture that encourages change. Mostly, when managers have to assess the success or failure of the initial assimilation of new technologies, they often ignore the influence of organisational culture (Fakhri et al., 2020). Many conceptualizations point to the possibility of different explanatory variables that can characterise an organisation's culture (Kanwal et al., 2019). Nevertheless, the acceptance of new technologies can influence the organisational culture. Pradana et al. (2020) stated that it is essential to find a match between the new technology system and organisational culture to facilitate companies in obtaining the potential benefits expected by the system. However, what is the crucial factor in organisational culture to shape digital transformation, particularly in the digital realm? This research suggests the digital conceptualisation of organisational culture.

As a conclusion from the various arguments above, it can be concluded that the use of digital technology cannot guarantee the success of its implementation (Lartey et al., 2021). Devi et al. (2021) suggest that when a technological system clashes with the organisation's culture, its implementation will be rejected in one of two ways—the system will be rejected or modified to fit the current culture. Furthermore, Abbasi et al. (2021) said that some believed that culture could be intentionally arranged and shaped. Indeed, practitioners advertise the digital culture required for digitalisation as an adaptive organisational culture (Roper et al., 2017). Therefore, digital culture is a means for organisations to plan digital strategies in a rapidly changing environment (Zhang et al., 2021). Thus, existing organisational cultures should encourage the challenge of accepted values and norms as a first step to considering whether digitalisation initiatives can be applicable to the company or organisation.

Notably, the organisations need to identify the existing cultural attributes and then select the attributes that can accelerate digital business and can also form new organisational, cultural attributes to support the success of digital business (Martínez-Caro et al., 2020). Therefore, companies need to be more proactive in reinterpreting corporate culture around the digital aspects of their workplace. According to Fakhri et al. (2020), a digital leader is those who play a role in determining the digital strategy and who is able to clearly combine digital cultures which are ready to drive transformation. Therefore, a certain digital culture is considered a prerequisite for success in developing the digitalisation of businesses. However, Martínez-Caro et al. (2020) cited that each company has a different level of success when it comes to the deeper use of digital technology.

### Digital Organisational Culture as an Antecedent

Previous work has shown that Information and Communication Technology (ICT) not only creates value, but also needs to be part of a business value creation process that aligns with the synergies of organisational factors (Farinha et al., 2016). Therefore, a better understanding of ICT needs to be developed, especially related to the reasons why companies adopt digital technology and how to use it. Achievement of value from digital technology is a new challenge faced by companies that have developed appropriate implantation and acceptance of technology, mostly in the aspect of organisational value.

Digital culture can also exist when the organisation plans for digital strategies in a rapidly changing environment (Martínez-Caro et al., 2020). Therefore, the existing organisational culture should encourage accepted values and norms even though they emerge as challenges (Pradana et al., 2020). It has to be settled before determining whether the initiative of digitalisation can be relevant. Roughly, the organisation should identify the attributes of the existing culture first before reducing cultural attributes that hamper business digitalisation (Martínez-Caro et al., 2020). At the same time, it is also essential to establish organisational culture attributes that support successful business digitalisation.

Most existing research focuses on adoption decisions and measuring the “intention to adopt.” However, it is essential to study post-adoption value variations in depth. It is crucial to examine the diffusion of digital technology as a multi-stage process that begins at adoption and extends to value creation (Zhang et al., 2021). Therefore, the development of the value of digital technology (DTVD) can be considered the ability of companies to create value through digital technology (Martínez-Caro et al., 2020). This conceptualisation is a reflection of the idea of describing how much companies need to invest in digital technology to be used in work activities. This includes new knowledge to innovate, ease of decision making, improving service quality for customers, improving coordination with suppliers, and redefining processes for maximum efficiency (Fakhri et al., 2020).

This digital culture needs to encourage the development of collaborative work values, such as creativity and innovation, challenges and initiative, and also permanent improvement through a shared digital strategy (Martínez-Caro et al., 2020). We believe that those existing literatures show that digital organisational culture has also emerged as a current phenomenon in the professional environment. It aligns with our plan to explore this study in Indonesia, since digitalisation has become one major strategy of Indonesian companies, both state-owned or private ones (Hasbi et al., 2020). As a phase to structure our research model, two hypotheses explaining the cultural events of digital organisations in DTVD are proposed:

H1. Digital organisational culture has a positive effect on business digitalisation.
H2. Digital organisational culture has a positive effect on digital organisational value.

### The Roles of Business Digitalisation and Digital Organisational Value

Business digitalisation supports access to knowledge residing within the organisation and also provides access to external knowledge (Pradana et al., 2020). Thus, it is clear that digital value is created from the support of business digitalisation (Martínez-Caro et al., 2020). Organisations often have difficulties in making changes in their culture only by digitising everything (Enkel et al., 2017). On the other hand, organisational culture keeps evolving and can take advantage of digital approaches to reinforce new behaviour in formal and informal ways. By doing this, the organisation can develop a digital culture not only among managers but among all company employees (Martínez-Caro et al., 2020).

The development of digital organisational value depends on business digitalisation because companies cannot exploit knowledge without first acquiring it (Cohen and Levinthal, 1990). In this regard, the digitalisation of business accommodates access to a broader knowledge that allows the company to increase its efficiency for digital organisational value. For example, a virtual community that operates through a website or social network allows companies to gather large amounts of information from community members. According to Ruiz-Ortega et al. (2018), big data collection will generate value through analysis and synthesis processes to be used to identify valuable information. With this, companies can determine patterns to draw conclusions about users and make decisions based on the information obtained.

The self-referential nature of digital technology means that innovative digital initiatives through widespread deployment require all-around access to digital tools (Birasnav et al., 2019). For example, Roper et al. (2017) studied how different types of digital infrastructure (data access systems and network connectivity) increase the impact of deep external knowledge on a company's digital organisational value. According to Devi et al. (2021), the broader the digital technology, the more likely organisations will create valuable and sustainable digital technology capabilities, thus contributing to value creation. Therefore, it is a challenge for academics in proving whether the digital aspects and their adoption yield a creation or alteration in organisational value, which eventually affects overall performance. To achieve that aim, we formulate the following hypotheses:

H3. Business digitalisation has a positive effect on digital organisational value.
H4. Business digitalisation has a positive effect on organisational performance.

### Organisational Performance as Outcome

Previous researchers in several countries have researched the influence of organisational culture on employee performance. Enkel et al. (2017) researched the influence of organisational culture on the performance of multinational company employees. The research results found that organisational culture had a significant positive effect on employee performance. Research into organisational culture on the performance of employees by Farinha et al. (2016) shows a positive influence of organisational culture on employee performance. The digital era has indeed explored the possibility of technologies in improving organisational performance. However, the effect of digital technologies tends to vary (Pradana et al., 2020). Therefore, there has been a demand or pressure in proving that digital technologies have a consistent positive effect on organisational performance (Fakhri et al., 2021). This paper focuses on the fact that digital technologies can have an effect on performance in two ways, which are from the perspective of business digitalisation and digital organisational value. Business digitalisation can benefit organisations by increasing connectivity and saving costs (Martínez-Caro et al., 2020). On the other hand, digital organisational value can also bring benefits by keeping employees' connectivity on the Internet while working on tasks. For sure, digital organisational value changes the ways to interact, communicate, send, and receive information within and across firms (Farinha et al., 2016).

Fakhri et al. (2020), with their research on the effect of organisational culture research on employee performance, generates a significant positive influence between organisational culture and employee performance. Another case with Aboazoum et al. (2015), who conducted a research on employee performance in Libya, who said that the organisational culture has a positive and significant relationship on employee performance. Ullah et al. (2022), in their research in the banking sector in Pakistan, stated that organisational culture has a significant positive effect on employee performance.

Uddin et al. (2018) researched Bangladesh's telecommunications sector and proved that organisational culture significantly affects employee performance. Nazir and Zamir's (2015) study stated that organisational culture has a significant positive influence on employee performance in various organisations in Pakistan. Omoregbe and Umemezia (2017) conducted research on the Nigerian banking sector, proving that organisational culture positively affects employee performance. Nguyen (2021) proves that organisational culture positively affects employee performance. However, a research by Fakhri et al. (2020) states that organisational culture negatively affects employees' performance, which has been proven with employees of state-owned enterprises. We would like to see whether the application of this research also works among Indonesian companies, thus we hereby construct these following hypotheses:

H5: Digital organisational value has a positive effect on organisational performance.
H6: Digital organisational culture has a positive effect on digital organisational value through a mediating effect of business digitalisation.
H7: Business digitalisation has a positive effect on organisational performance through a mediating effect of organisational value.

**Figure 1:**
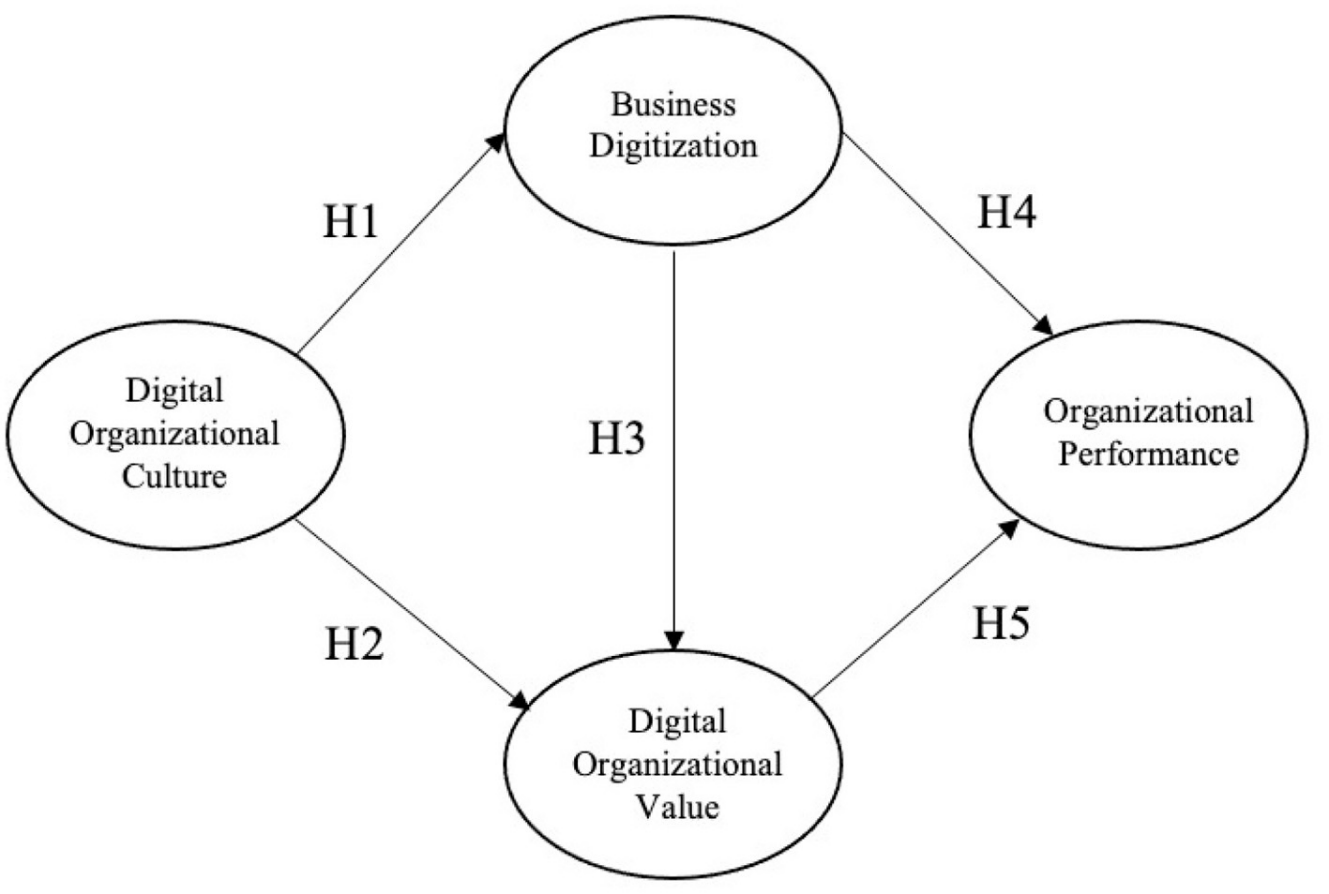
Research model (adopted from Martínez-Caro et al., 2020).

We visually present the research framework as seen in Figure 1.

## Research Methodology

We use a sample of Indonesian state-owned companies to conduct our analysis. From the 500 online questionnaires we distributed, we received responses from 263 managers of state-owned companies in Indonesia (65.75% response rate). The low response rate was due to the reluctance of several companies to participate in our survey. However, we believe the 263 willing respondents were decent representations because they are managers in digitised companies. This study uses multiple constructs that are measured by a multi-item scale. To complete the validity and acceptance of the data, we used several steps which started with interviewing 25 top managers to conclude as a pre-test measuring tool. Respondents were asked to revise the survey for the sake of clarity of the questions and determine the appropriate size of the scale. After that, we made improvements to each item of the question before distributing the questionnaire.

The measures we used for this study are variables previously introduced by Martínez-Caro et al. (2020). We conducted a preliminary study of the dimensions of the scale through exploratory factor analysis. Partial least squares are used in this study to measure the model based on the principal of the component-based estimation approach (Chin et al., 2013). We then analysed the data using software SmartPLS version 3.0.

## Result and Discussion

A measurement model is a model that connects latent variables with manifest variables. In this study, there were 4 latent variables which were shaped by indicators as formative constructs and measured by 13 indicators. Here are the results of convergent validity tests that include loading factors and AVE values on each research variable.

Based on the processing of results presented in Table 1, we conclude that not all of the indicators have loading factors >0.707, which is the cut-off validity value (Hair et al., 2016). It has made two indicators invalid, namely BD1 and DOC1 (indicated by ^*^). After removing all invalid indicators, we ran the data again and concluded some values as seen in subsequent tables.

**Table 1:** Convergent validity test.

**Variable**	**Indicators**	**Loading factor**
Business digitalisation	BD1	0.034^*^
	BD2	0.886
	BD3	0.764
Digital Organisational culture	DOC1	0.406^*^
	DOC2	0.831
	DOC3	0.873
	DOC4	0.885
Digital Organisational value	DTV1	0.775
	DTV2	0.811
	DTV3	0.874
Organisational performance	OP1	0.799
	OP2	0.878
	OP3	0.717

*(*

* *) = not included in further calculation*.

*Internal consistency reliability* is used to measure how much the indicator variable increases when the latent variable increases as well. The criteria used are *Composite Reliability* (CR) and also *Cronbach's Alpha* (CA). We also measured the Harman's single factor (HSF) test to check the method bias. Our result shows that the CMB was clearly non-existent as the total estimated variance was just 31.67%, which is lower than the cutoff value of 50% (Hair et al., 2016).

Based on Table 2, we see that all latent variables have a *Composite Reliability* (CR) value and a *Cronbach's Alpha* (CA) of more than 0.7, which makes them reliable. This shows that all indicators have consistency in measuring each construct. The *Cronbach's Alpha* indicator exceeded the recommended threshold of 0.7 (Nunnally and Bernstein, 1994), the composed reliability coefficient was >0.7 (Anderson and Gerbing, 1988), and the average variance extracted (AVE) was over 0.5. By implementing the Fornell Larcker criterion, the AVE scores of a construct should be lower than the shared variance for all model constructs. From the results of the study, the AVE scores of every construct are lower than that it's shared variance (Fornell and Larcker, 1981) (Table 3).

**Table 2:** Reliability test.

	***Cronbach's Alpha***	***Composite Reliability***	**AVE**
Business digitalisation	0.902	0.939	0.720
Digital organisational culture	0.776	0.844	0.832
Digital organisational value	0.925	0.947	0.764
Organisational performance	0.884	0.928	0.725

**Table 3:** Fornell and Larcker test.

	**Business digitalisation**	**Digital organisational culture**	**Digital organisational value**	**Organisational performance**
Business digitalisation	0.788			
Digital organisational culture	0.467	0.810		
Digital organisational value	0.557	0.543	0.851	
Organisational performance	0.199	0.380	0.632	0.912

We also present the Heterotrait–Monotrait ratio (HTMT) in Table 4. This approach is used to assess discriminant validity, where—in our study—all values were lower than the threshold of 0.9 (Henseler et al., 2016).

**Table 4:** Heterotrait–Monotrait ratio of correlations.

	**Business digitalisation**	**Digital organisational culture**	**Digital organisational value**
Business digitalisation			
Digital organisational culture	0.650		
Digital organisational value	0.454	0.392	
Organisational performance	0.241	0.266	0.132

In the next part, we analysed the structural model. A structural model connects exogenous latent variables with endogenous latent variables or endogenous variable relationships with other endogenous variables. After analysing the path coefficient, we found that digital organisational culture (DOC) with path coefficients of 0.451 and 0.436 affects business digitalisation and digital Organisational Value. Then, business digitalisation and digital organisational value both positively affect organisational performance with track coefficients of 0.309 and 0.050. Therefore, H1, H2, and H4 are accepted. The only relationship that contains a negative value is business digitalisation which turns out to be unrelated to digital organisational valueThis can be seen from the negative coefficient value, which is at −1,101. The coefficient numbers from the smartPLS software can be seen in Table 5.

**Table 5:** Summary of statistical test results.

**Hypothesis**	**Path**	**Path coefficient**	***P*-values**	**VIF**	**Decision**
H1	Digital organisational culture -> business digitalisation	0.451	0.000	2.178	H1 accepted
H2	Digital organisational culture -> digital organisational value	0.436	0.002	2.344	H2 accepted
H3	Business digitalisation -> digital organisational value	−1.101	0.176	2.215	H3 rejected
H4	Business digitalisation -> organisational performance	0.309	0.000	2.546	H4 accepted
H5	Digital organisational value -> organisational performance	0.050	0.017	2.003	H5 accepted
H6	Digital organisational culture -> digital organisational value with mediating effect of business digitization	−1.651	0.212	1.914	H6 rejected
H7	Business digitalisation -> organisational performance with mediating effect of digital organisational value	0.253	0.117	4.103	H7 accepted

## Conclusion

After finalising the results, we have come to a conclusion. We develop this research by exploring a proposed model involving digital organisational culture with the final goal to enhance organisational performance. We conclude that digital organisational culture can become an essential factor in improving digital strategy and performance.

First, the lack of a clear business digitalisation strategic direction often leads to conflicting organisational values. Therefore, business digitalisation does not really affect digital organisational values which can be seen from our research results. This also applies for either our direct and indirect effect. As previous research also suggested, we also agree that committing to the digital culture is required for digitalisation as an adaptive organisational value (Roper et al., 2017). Although a digitalised organisational culture can lead to better performance, especially in an environment already familiar with digital technology, the ideal digital organisation value cannot be achieved without a clear digitalisation strategy. This is proven by our empirical results of indirect effects. The organisations need to identify the existing cultural attributes then select the attributes that can accelerate digital business and form new organisational cultural attributes to support the success of digital business (Martínez-Caro et al., 2020).

Furthermore, the development of digital organisational value still depends on business digitalisation because companies cannot exploit knowledge without first acquiring it (Birasnav et al., 2019). The impact of deep external knowledge on a company's digital organisational value had a significant positive effect on employee performance.

From a managerial perspective, this study provides insight for managers regarding the significant role of a digital organisational culture in facilitating business digitalisation and the development of IT value. However, the lack of a clear digital strategic direction often leads to constraints and difficulties in achieving work synergy. We believe this case relies on the managers' role in identifying the attributes of the existing culture and minimising unnecessary cultural attributes that prevent or slow down digitalisation.

### Limitation and Suggestions for Future Research

Cultural attributes that prevent or slow down digitalisation need to be reduced and the ones supporting successful digital technology exploitation must be maintained and exploited. In Indonesia, a country where business digitalisation is rapidly developing, top management personnels should preserve conventional organisational culture and transform it into a digital environment. Therefore, it can leave room for more studies in the future. Upcoming research can involve more dimensions of culture or explore other theories of business digitalisation.

Although we are quite satisfied with the outcome of our research, we also acknowledge some limitations to our work. First, due to limited available information, our study does not include all of the variables that explain performance. Second, we must not rush in generalising the results because the research was conducted in various Indonesian state-owned companies with different characteristics. However, the limitation also opens further opportunities for future studies by focusing on one specific business sector.

## Data Availability Statement

The original contributions presented in the study are included in the article/supplementary material, further inquiries can be directed to the corresponding author/s.

## Author Contributions

AS and MP wrote the initial draft, analysed the data, and operated the software. SS and RR conducted field research and wrote the final draft. All authors contributed to the article and approved the submitted version.

## Conflict of Interest

The authors declare that the research was conducted in the absence of any commercial or financial relationships that could be construed as a potential conflict of interest.

## Publisher's Note

All claims expressed in this article are solely those of the authors and do not necessarily represent those of their affiliated organizations, or those of the publisher, the editors and the reviewers. Any product that may be evaluated in this article, or claim that may be made by its manufacturer, is not guaranteed or endorsed by the publisher.
